# American Medical Association—Session for 1869

**Published:** 1869-06-15

**Authors:** 


### CHICAGO MEDICAL JOURNAL.

Vol. XXVI.—JUNE 15, 1869.—No. 12.

### REPORTS OF MEDICAL SOCIETIES.

### *American Medical Association—Annual Session for 1869.*

[Concluded from last Number.]

The Committee on Nominations—Dr. J. J. Woodward, U. S. N., President—reported the following names:

REPORT OF THE NOMINATING OCMMITTEE.

NEW ORLEANS, LA., May 6, 1869.

For President—Geo. Mendenhall, Ohio.

For Vice Presidents—Warren Stone, La.; Lewis A. Sayre, N. Y.; F. Gurney Smith, Penn.; John S. Moore, Mo. follows:

The Committee on Nominations unanimously report as follows:

For Assistant Secretary—Wm. Lee, D. C.

For Treasurer—Caspar Wister, Penn.

For Librarian—Robt. Reyburn, D. C.

Committee of Arrangements—Thomas Antisell, Chairman; Robert Reyburn, C. M. Ford, L. W. Ritchie, W. J. C. Duhamel, D. R. Hayner, C. F. Nally.

Committee of Publication—F. Gurney Smith, Pa., Chairman; W. B. Atkinson, Pa.; A. J. Semmes, Ga.; Robert Reyburn, D. C.; Caspar Wister, Pa.; H. F. Eskew, Del.; Wm. Maybury, Pa.

Committee on Medical Literature—J. J. Woodward, U. S. A., Chairman; W. H. Anderson, Ala.; Theophilus Parvin, Ind·; Hosmer A. Johnson, Ill.; C. W. Parsons, R. I.

Committee on Prize Essays—Grafton Tyler D. C., Chairman N. R. Lincoln, D. C.; N. R. Smith, Md.; G. W. Miltenberger, Md.; W. R Dunbar, Md.

Committee on Epidemics—Add the following to fill vacancies: J. K. Bartlett, Wis.; J. B. Jackson, Ky.

Committee on Education— T. G. Richardson, La., Chairman; E. W. Jenks, Mich.; E. S. Gaillard, Ky.; W. M. McPheeters, Mo.

Time for meeting, in Washington, first Tuesday in May 1870.

J. J. WOODWARD, U. S. A., *Chairman.*

The report was unanimously adopted.

Dr. Davis offered the following:

*Resolved*, That a special committee of three be appointed by the President to present copies of the resolutions adopted before the several State medical societies at as early a period as possible. Adopted.

Dr. Chaille, of Louisiana, chairman of the committee presented a report on medical nomenclature, which was received and adopted, and referred to the Committee on Publication.

The Committee on Nomenclature of Diseases have the honor to report that it has examined the "Provisional Nomenclature of the Royal College of Physicians" of London, and is of the opinion that it is desirable for this association to recommend and adopt the same for general use for this country, with such modifications as, on deliberate consideration, may appear to be necessary. The following resolutions are therefore submitted.

1. *Resolved*, That a special committee of fifteen be appointed by the President to take this subject into deliberate consideration, and to report at the next annual session what alterations, if any, are necessary to adapt the proposed nomenclature to general use in the United States.

2. That this committee be authorized to fill up any vacancies which may occur upon it.

3. That The Committee on Publication be authorized to publish for general distribution one thousand copies of the English and Latin portions of this nomenclature, under the designation of the Proposed Nomenclature, prefacing the same with such remarks as may be deemed necessary to secure the criticism and co-operation of as large a number of American medical men as practicable.

4. That the committee hereby appointed be directed to draw the attention of the Surgeon General of the Army, of the Chief of the Bureau of Medicine and Surgery of the Navy, and of the Superintendent of the Census, to the question of their official adoption of the proposed Nomenclature; to invite them to appoint whom they see fit to represent them on this committee; and to solicit such co-operation as may be necessary to accomplish the purpose desired viz., the final adoption of nomenclatureand classification as will receive the conjoint approval of the official medical bureaus of the Government and or the general profession.

STANFORD E. CHAILLE, M. D., *Chairman.*

COMMITTEE.

S. E. Chaille, Louisiana;
J. J. Woodward. United States Army:
A. B. Palmer, Michigan;
F. F. Smith, Pennsylvania;
J. F. Heustis, Alabama;

The following committee of fifteen was appointed:

Francis G. Smith, Chairman; J. J. Woodward, U. S. A.; R. F. Michel, Alabama; A. B. Palmer, Michigan; S. E. Chaille, Louisiana; L. P. Yandell, Jr., Kentucky; Austin Flint, New York; Alonzo Clark, New York; George B. Wood, Pennsylvania; S. H. Dickson, Pennsylvania ;E. Jarvis, Massachusetts; Theo. Parvin, Indiana; W. M. Mc-

Pheeters, Missouri; E. M. Snow, Rhode Island; N. PinckneyU. S. N.

The Committee on Medical Education having referred matters at issue to State Medical societies, Dr. E. S. Gaillard of Louisville, offered the following motion:

*Moved*, That the adoption of a uniform rate of collegiate's fees $120, being the MINIMUM, be accepted as the sentiment and desire of this association.

Dr. Gaillard stated that he would not trespass upon the time of the association in speaking upon this motion; that all of the members present were fully informed upon this subject. He said the profession desired to learn the wish and decision of the association upon this all important question, and he asked a full expression of opinion and a full vote in regard to it.

Dr. Sayre, of New York, opposed the resolution, but, on understanding that it did not prohibit an increase of fees, withdrew his objections. He spoke against cheap medical colleges, which allured young men to an imperfect medical education, who were afterward turned back to the plow.

An amendment was proposed by Dr. Logan, of Louisiana, to make the minimum $140 instead of $120.

Dr. Mussey, of Ohio, opposed the amendment, and stated that the fees of Ohio had to be kept down, to accord with the fees of Michigan colleges. The location of the college and the cost of living made the difference. A hundred and forty dollar college is considered good, simply because a hundred and forty dollars is the fee, while other colleges were equally as good where the fees were only eighty dollars. It is impracticable to accomplish this change at once. A new college starts and comes up to the full standard of the old college in the vicinity, and the latter comes down with its fees. The new college must come down also, in order to maintain itself.

Dr. McPheeters, of Missouri, did not agree with Dr. Mussey that it was impracticable to fix the collegiate fees at a minimum of one hundred and twenty dollars. He favored the original resolution, without amendment.

Dr. Palmer, of Michigan, alluded to the remarks of Dr. Sayre, of New York, disparaging one-horse and cheap colleges. The University of Michigan was established and allowed a donatian from the General Government of two townships of land, and it has husbanded its resources, and can maintain itself with moderate fees. Under the organic law of the State, citizens of Michigan were entitled to the benefits of the university free of charge, and, as a liberal donation had been made by the General Government, students had been admitted from other States on the same terms. Lately, however, a small fee had been charged for students from other States who received the benefits of the lectures. We come up in fees just as far as we think is for the advantage of the institution, and we do not go beyond that point, beeause it will diminish the numbers. We are willing to put up the fees for students from other States to one hundred and forty dollars, if neighboring States will make the same requirements as we do.

Dr. Palmer then commenced describing the *great advantages of this school*, when Dr. Gaillard called him to order, stating that we were present to discuss principles involved, and not to listen to eulogies upon special schools.

Dr. Davis, of Illinois—I do not object to discuss the fees, but I do claim that it is out of place to advertise the superior claims of State colleges here. We have had no more illiterate students in our Illinois college than have come to us after one course in the University of Michigan.

Dr. Parvin, of Indiana—I move to amend by striking out $140 and inserting $100. If we make the fees of colleges uniform, the next step will be to make the fees of practitioners uniform—the same in villages of the West as in the city of New York—and that is not equitable or practicable.

Dr. Logan, of Louisiana, advocated the sum of $140 as the minimum of college fees, and advocated the adoption of his amendment. Lost.

The amendment of Dr. Parvin, to fix the minimum at $100, was also lost.

The resolution was then, as originally presented, unanimously adopted.

Special committee on the relative advantages of Syme's and Pirogoff's mode of amputating at the ankle—Dr. G. A. Otis, U. S. A., chairman; Dr. J. M. Holloway, of Louisville, Ky.

Proposed by J. J. Woodward. Approved.

Dr. Bemiss presented from Dr. John Waters, of St. Louis, Mo., a paper on the Doctrines of Force—Physical and Vital.

Dr. Toner, D. C., moved that a Committee on Variola be appointed—Dr. J. Jones, chairman. Adopted.

Dr. Pinckney, U. S. N., made statements concerning relative grades of rank. The paper was ordered to be spread upon the minutes.

Association adjourned to meet at 9 o'clock A. M., Friday, May 7th.

FOURTH DAY, FRIDAY, MAY 7, 1869.

The association met at 9 o'clock, Dr. Baldwin in the chair.

Reading of the minutes omitted.

Dr. Joseph Jones, Louisiana, presented a number of specimens of pathology, anatomy, and natural history. The illustrations were very interesting, and received with applause.

On motion of Dr. Garrish, Kentucky, the thanks of the association were tendered to Dr. Jones.

On motion of Dr. F. G. Smith, of Pennsylvania, the following resolution was unanimously adopted by a vote of the members present, standing as a mark of respect:

*Resolved*, That the thanks of this association are justly due and are hereby tendered to the President, for the uniform kindness and courtesy with which he has presided over its deliberations, and to the Committee of Arrangements, the physicians, and the citizens of New Orleans, for the generous hospitality and fraternal kindness with which we have been received and treated during our sojourn in their city, with the assurance that the memories of this visit will always be among the brightest and most enduring of our lives.

*Resolved*, That we also present our thanks to the various railroad and steamboat companies who have so liberally extended to us facilities of transportation, and to the daily press for their efficient aid in reporting the proceedings of this meeting.

On motion of J. P. Moore, of Mississippi, the following preamble and resolution were adopted:

WHEREAS the contract system is contrary to medical ethics.

*Resolved*, That all contract physicians, as well as those guilty of bidding for practice at less rates than those established by a majority of regular graduates of the same locality, be classed as irregular practitioners.

The following reports of the sections followed.

Sections on Meteorology, Medical Topography and Epidemics reported. Paper accepted and referred to the Committee on Publications.

Section on Practical Medicine and Obstetrics reported and were accepted, and referred to Committee on Publication.

And the report on Training of Nurses was accepted and the resolutions adopted.

Section on Medical Jurisprudence, Hygiene, and Physiology reported. Committee continued for next year. Report accepted and referred to Committee on Publication.

Section on Surgery proposed that their report be received without formality and be referred to the Committee on Publication. Adopted.

After being read, the report was accepted and ordered to be published.

Section on Psychology, the same disposition.

The President appointed Dr. J. M. Toner a committee of one, at Washington, D. C., to assist the Librarian of Congress to keep the books of the Association.

On motion for adjournment, the President delivered this address, which was unanimously accepted and ordered to be published in the transactions of the Association.

GENTLEMEN—Before I submit the motion just made, and which, when adopted, will practically close my official relations to this body, allow me to return you my most cordial and grateful thanks for the universal kindness which I have received at your hands. Whatever my future lot in life may be, the world holds no honors which to me can equal those conferred by you. The fraternal good-will which has so conspicuously marked your deliberations has been to me a matter of infinite satisfaction and pride, and will not be the least among the grateful memories which will gladden my heart as I may hereafter review the incidents of my official connection with you.

To win your judgment and approval, to hold up the dignity of fellowship, the usefulness of association and the interest and prosperity of the profession at large have certainly occupied my most anxious thoughts since my elevation to this position; yet to cherish and promote the intimate and cordial relations of friendship between the individual members of this Association against all sectional distinctions or geographical lines, has also been among the chief objects of my ambition and the earnest desires of my heart. Could I now believe that my efforts have contributed in the slightest degree to enlarging that harmony of sentiment and fraternal feeling which has been so apparent throughout this meeting, I should feel that I had commenced at least to make some return for the great honor and kindness received at your hands.

It now only remains for me, gentlemen, to again express to you my thanks, to wish you a safe return to your homes and labors, a happy reunion with your friends and families, and to pronounce that sad word, over which the heart of friendship would fain linger, as I bid you an affectionate farwell. W. O. BALDWIN, *Pres't A. M. A.*

The Convention adjourned to meet in Washington, D. C., the second Tuesday in May, 1870.

